# Hormone-Sensitive Lipase Knockouts

**DOI:** 10.1186/1743-7075-3-12

**Published:** 2006-02-10

**Authors:** Fredric B Kraemer, Wen-Jun Shen

**Affiliations:** 1 VA Palo Alto Health Care System, Palo Alto, CA and Division of Endocrinology, Department of Medicine, Stanford University, Stanford, CA, USA

## Abstract

All treatments for obesity, including dietary restriction of carbohydrates, have a goal of reducing the storage of fat in adipocytes. The chief enzyme responsible for the mobilization of FFA from adipose tissue, i.e., lipolysis, is thought to be hormone-sensitive lipase (HSL). Studies of HSL knockouts have provided important insights into the functional significance of HSL and into adipose metabolism in general. Studies have provided evidence that HSL, though possessing triacylglycerol lipase activity, appears to be the rate-limiting enzyme for cholesteryl ester and diacylglycerol hydrolysis in adipose tissue and is essential for complete hormone stimulated lipolysis, but other triacylglycerol lipases are important in mediating triacylglycerol hydrolysis in lipolysis. HSL knockouts are resistant to both high fat diet-induced and genetic obesity, displaying reduced quantities of white with increased amounts of brown adipose tissue, increased numbers of adipose macrophages, and have multiple alterations in the expression of genes involved in adipose differentiation, including transcription factors, markers of adipocyte differentiation, and enzymes of fatty acid and triglyceride synthesis. With disruption of lipolysis by removal of HSL, there is a drastic reduction in lipogenesis and alteration in adipose metabolism.

## Introduction

Free fatty acids (FFA) are a major energy source for most tissues in mammals. Circulating FFA in plasma are primarily derived from adipose tissue, which is the main repository for the storage of triacylglycerol. All treatments for obesity, including dietary restriction of carbohydrates, have a goal of reducing the storage of fat in adipocytes. The chief enzyme responsible for the mobilization of FFA from adipose tissue, i.e., lipolysis, was thought to be hormone-sensitive lipase (HSL). This review addresses some aspects of the organization of components of fat metabolism in the adipocyte, lipolysis and lipogenesis, and how genetic manipulation of the pathway of lipolysis affects fat metabolism in the adipocyte.

## Properties of HSL

HSL is an intracellular, neutral lipase that has broad substrate specificity, catalyzing the hydrolysis of triacylglycerol, diacylglycerol, monoacylglycerol, and cholesteryl esters, as well as retinyl esters; however, it possesses no phospholipase activity [1]. Its activity against diacylglycerol is about 10-fold and 5-fold higher than its activity against triacylglycerol and monoacylglycerol, respectively, whereas its activity against cholesteryl esters is about twice its activity toward triacylglycerol. The hydrolytic activity of HSL against triacylglycerol and cholesteryl esters, but not against diacylglycerol, is stimulated by phosphorylation mediated primarily by protein kinase A (PKA) [1].

## Lipolysis

**Figure 1:**
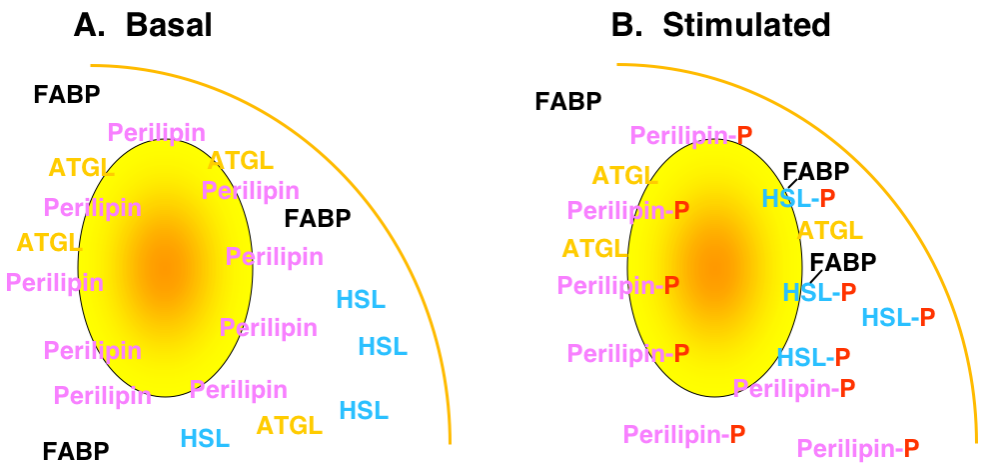
**Cartoon model of lipolysis**. Under basal conditions perilipin is localized to the lipid droplet, along with other droplet associated proteins, such as ATGL (adipose triglyceride lipase), whereas HSL is primarily localized in the cytosol along with other cytosolic proteins such as FABP (fatty acid binding protein). Following lipolytic stimulation, PKA is activated, resulting in the phosphorylation of perilipin and HSL. This is associated with the translocation of HSL from the cytosol to the lipid droplet where hydrolysis of the triacylglycerol lipid droplet occurs.

The regulation of lipolysis is complex and, although not completely understood, involves multiple mechanisms, including lipolytic (β-adrenergic agonists, ACTH, etc.) and anti-lipolytic (insulin, adenosine, etc.) hormones and their cognate receptors and signaling pathways, particularly involving cyclic AMP and PKA. Current working models for the mechanisms underlying lipolysis have focused on steps downstream of hormone receptors and signaling cascades, concentrating on lipid droplet-associated proteins, such as perilipins, and lipases, such as HSL and others, that appear to play vital roles in lipolysis [1]. In a simplified view (Figure 1), these models suggest that, under basal, unstimulated conditions, perilipin decorates the surface of the lipid droplet, protecting the lipid droplet from hydrolysis by HSL, which is primarily located within the cytosol. Upon lipolytic stimulation, PKA is activated, resulting in the phosphorylation of both perilipin and HSL. Phosphorylation of perilipin then facilitates the translocation of HSL from the cytosol to the lipid droplet, where hydrolysis of triacylglycerol and lipolysis can proceed.

Studies of mice where HSL has been inactivated by homologous recombination have provided important insights into the functional significance of HSL and into adipose metabolism in general. HSL null mice have a normal physical appearance and are nonobese, lacking an apparent phenotype, with the exception that homozygous males have severe oligo- or azospermia and are infertile [2]. However, careful examination of adipocytes does reveal abnormalities. There are histological changes in adipose tissue. Brown adipose tissue (BAT) displays increased cell size and white adipose tissue (WAT) displays an increased heterogeneity of cell size, with both larger and smaller cells being more prevalent [2,3]. Examination of enzyme activity showed the complete absence of neutral cholesteryl ester hydrolase activity in adipose tissue, both WAT and BAT. Moreover, examination of other tissues where HSL is expressed, for instance adrenal, testis, ovary, and heart, showed the almost complete absence of neutral cholesteryl ester hydrolase activity [2,4]. Thus, HSL is responsible for all, or practically all, of the neutral cholesteryl ester hydrolase activity in adipose and steroidogenic tissues. In contrast, triacylglycerol lipase activity in WAT was reduced by only 40–50% and triacylglycerol lipase activity in BAT was similar to wild type mice [2,3,5], suggesting the existence of one or more other neutral triacylglycerol lipases. Measurement of plasma concentrations of glycerol and FFA showed a 40–60% reduction in circulating levels under unstimulated conditions, whereas there was a small, but markedly attenuated, increase following isoproterenol injection, supporting an important, but not exclusive, role for HSL in lipolysis. *In vitro *studies of adipose cells isolated from HSL null mice recapitulated the *in vivo *findings for the most part; there was a marked defect or complete absence of catecholamine-stimulated glycerol release in adipose cells from HSL null mice, whereas catecholamine-stimulated FFA release was still observed, but attenuated [2,3,5,6]. This apparent discrepancy in the release of glycerol and FFA from adipose cells of HSL null mice has been clarified by the observation that diacylglycerol content increased markedly in adipose tissue of HSL null mice [5]. In both white and brown adipose tissue from HSL null mice, catecholamine-stimulation caused the release of small amounts of FFA without any stimulated glycerol release, and a marked accumulation of diacylglycerol [5]. These results are consistent with the known substrate specificity of HSL being 10-fold higher against diacylglycerol than triacylglycerol. Indeed, direct measurement of diacylglycerol lipase activity showed that activity was reduced >95% in adipose tissue of HSL null mice, whereas diacylglycerol lipase activity was unaltered in muscle and liver [7]. Therefore, HSL, though possessing triacylglycerol lipase activity, appears to be the rate-limiting enzyme for cholesteryl ester and diacylglycerol hydrolysis in adipose tissue and is essential for complete hormone stimulated lipolysis.

## Lipases in adipocytes

The presence of residual triacylglycerol lipase activity in adipose tissue of HSL null mice has led to the search for other lipases responsible for the maintenance of the mobilization of FFA from fat. Using functional proteomics and oleic acid-linked agarose chromatography, Soni et al [8] identified carboxylesterase 3, also known as triacylglycerol hydrolase, as an abundant lipase in adipose tissue that continues to be expressed in HSL null mice. Using an *in silico *approach, Zimmermann et al [9] identified a protein that possessed lipase activity and was highly expressed in adipose tissue which they termed adipose triglyceride lipase (ATGL). ATGL had been identified by other authors, who termed it desnutrin [10] and calcium independent phospholipase A_2_ζ [10,11]. ATGL contains a patatin-like sequence and has homology with several other patatin-like family members such as adiponutrin and GS2 or iPLA_2__η_ [11]. Based on immunoprecipitation of ATGL from adipose tissue homogenates, ATGL was reported to account for 60–70% of the neutral triacylglycerol lipase activity in both wild-type and HSL null mice [9]. However, it is apparent that there are multiple lipases in adipose tissue since 23 different proteins that potentially possess lipolytic or esterolytic activity in mouse adipose tissue have been identified using a proteomics approach that took advantage of fluorescent tags that mimic lipid substrates [12]. In unpublished work we have recently examined the relative expression levels of 11 of the lipase/esterases in WAT using quantitative RT-PCR analysis. The proteins examined are: HSL, ATGL, adiponutrin, triacylglycerol hydrolase, α/β hydrolase containing mRNA 5 (also known as CGI-58) and 11 (also known as Beuren-Williams Syndrome Critical Region 21), acylpeptide hydrolase, esterase 1, esterase D, carboxylesterase ML1, and membrane-associated calcium-independent phospholipase A_2_γ. We found the following relative expression levels in normal mouse WAT in descending order of abundance: ATGL, HSL, triacylglycerol hydrolase, adiponutrin, membrane-associated calcium-independent phospholipase A_2_γ, esterase D, CGI-58, carboxylesterase ML1, acylpeptide hydrolase, Beuren-Williams Syndrome Critical Region 21, and esterase 1. Since the residual basal triacylglycerol lipase activity in WAT of HSL null mice indicates the existence of other lipase(s), we examined the expression levels of these other 10 lipase/esterases in WAT of HSL null mice. Surprisingly, almost all of the 10 lipase/esterases showed decreased levels of expression when compared with wild-type mice, with ATGL and triacylglycerol hydrolase reduced to only 30% of that of wild-type mice. Thus, multiple proteins with lipase/esterase activity are expressed in WAT and the absence of HSL does not result in a compensatory increase in the mRNA of any of these proteins, but rather in a global decrease in the expression of all lipases/esterases.

## HSL KO mice are resistant to obesity

When the influence of HSL on the development of dietary induced obesity was examined by feeding high fat diets for 15 weeks, 20–26% lower weights were observed in male and female, respectively, HSL null mice compared with control [13]. This occurred even though there was a higher food intake per body weight in HSL null mice and without any evidence of lipid malabsorption. There was increased fasting induced weight loss and higher core body temperatures in HSL null mice, consistent with an increase in energy expenditure. Even though total body weights were similar on a normal chow diet, total WAT mass was reduced in HSL null mice compared to control and this difference was exaggerated on a high fat diet, with total WAT weight more than 70% lower in HSL null mice than in control. In contrast to WAT, interscapular BAT was larger in HSL null mice than in control. In addition to resistance to diet-induced obesity, the absence of HSL also protects against genetic obesity, based on the observation that mice homozygous for both HSL and leptin deficiency (*ob/ob*) are 26% lighter than *ob/ob *mice and this is reflected by a reduction in WAT mass [14]. The greater amount of heterogeneity of cell size in HSL null mice is exaggerated in mice with combined HSL and leptin deficiency, with a marked increase in small, lipid-devoid cells that appear to have characteristics of pre-adipocytes [14].

## Altered adipose gene expression in HSL KO mice

In addition to accumulation of diacylglycerol in tissues noted above, cholesterol content in adipose tissues was increased in HSL null mice and this was accentuated by high fat feeding where cholesterol content was 5-fold higher in HSL null mice [13]. Besides changes in histology and lipid content, HSL deficiency has multiple effects on adipose metabolism. Levels of mRNA expression of peroxisome proliferator-activated receptor γ (PPARγ) and CCAAT/enhancer binding protein α (C/EBPα), two of the major transcription factors for adipogenesis [15,16], were suppressed 40–70% in WAT of HSL null mice. Similarly, PPARα levels were also diminished ~70% in WAT of HSL null mice.

In parallel to the suppression of transcription factors, mRNA expression of markers of adipose differentiation such as adiponectin, leptin, resistin and adipsin, which are all humoral factors derived from adipose tissue, were decreased 60–90% [13]. In contrast, expression of TNFα was up-regulated 2–3-fold in WAT and 5–10-fold in BAT of HSL null mice compared with control. Again consistent with the reduction in adipogenic transcription factors, all genes associated with fatty acid and triglyceride metabolism, such as adipocyte fatty acid binding protein, perilipin, lipoprotein lipase, glycerol-3-phosphate acyltransferase (GPAT), mitochondrial GPAT, acetyl-CoA carboxylase, fatty acid synthase, acyl-CoA synthetase, acyl-CoA:diacylglycerol acyltransferase 1 (DGAT-1), DGAT-2, ATP citrate lyase, were reduced in WAT of HSL null mice on either a normal chow or high fat diet [13,17]. These alterations in gene expression resulted in a marked decrease in fatty acid esterification pathways and in the synthesis of neutral lipids and glycerol-phospholipids in WAT of HSL null mice [17]. Thus, with disruption of lipolysis by removal of HSL, there is a drastic reduction in lipogenesis, which could allow a more efficient export of fatty acids released by lipolysis out of the cell.

In accordance with the down-regulation of adipogenic transcription factors, expression of insulin receptor, insulin receptor substrate-1 and glucose transporter 4 mRNA was decreased 30–80% in WAT of HSL null mice compared with control [13]. However, examination of genes involved with cholesterol metabolism revealed that expression of 3-hydroxy-3-methylglutaryl-CoA (HMG CoA) reductase, the rate-limiting enzyme for cholesterol synthesis, was up-regulated ~2-fold; whereas, HMG CoA synthase-1, an enzyme upstream of HMG CoA reductase, was down-regulated 50–70% in WAT of HSL null mice [13]. Surprisingly, expression of acyl-CoA:cholesterol acyltransferase 1, the enzyme that mediates the esterification of cholesterol to cellular cholesteryl esters was increased 2–4-fold in WAT and 5–8-fold in BAT. Expression levels of the lipogenic transcription factor sterol regulatory element binding protein-1c (SREBP-1c) were suppressed 50–75%, whereas expression of SREBP-2, which controls cholesterol synthesis and uptake, was up-regulated 2-fold. In parallel to the changes in SREBP-2, uncoupling protein 2 was increased 3–4-fold in BAT in HSL null mice [13].

## Insulin and glucose metabolism in HSL KO mice

The effects of HSL deficiency on insulin and glucose metabolism is controversial in that several papers have been published that report conflicting results. For instance, overall insulin sensitivity in HSL null mice has been reported to be decreased by some authors [7] and normal by other authors [18,19]. Hepatic insulin sensitivity has been reported to be increased [18,19], whereas insulin sensitivity in adipose tissue and in muscle has been reported to be reduced [7,17] or normal [18,19]. Likewise, insulin secretion in HSL null mice has been reported to be either normal [7,20] or reduced [21,22]. The bases for these differences in results are not readily apparent, but may be due to differences in the genetic background on which the HSL null mice have been examined or may depend on whether the studies have been conducted *in vitro *or *in vivo*.

## Macrophages in adipose tissue of HSL KO mice

Recent observations that there is increased macrophage infiltration into WAT in obesity have been extended to HSL null mice [23]. Even though there is decreased WAT mass in HSL null mice as opposed to the increase seen in obesity, there is a greater prevalence of hypertrophied adipocytes in HSL null mice. Recent studies have observed that, in parallel to adipocyte hypertrophy, there is an increase in macrophages located in crown-like structures surrounding adipocytes with the macrophages scavenging adipocyte-free lipid droplets released from cells undergoing necrotic-like cell death [23]. Thus, the greater numbers of macrophages in WAT in HSL null mice could contribute to the increase in TNFα and some of the other changes in adipocyte function observed.

## Conclusion

**Figure 2:**
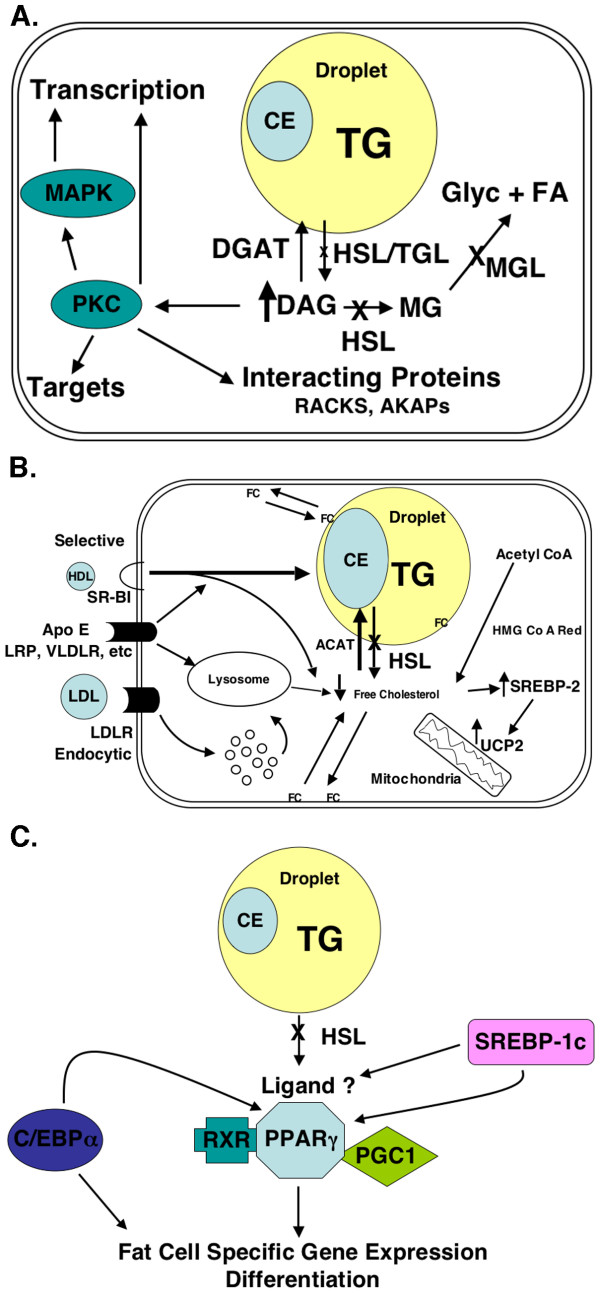
**Cartoon models of potential mechanisms underlying the altered adipocyte metabolism in HSL KO mice. Panel A. **Since HSL is the key diacylglycerol lipase in adipose tissue, diacylglycerol accumulates in HSL null mice leading to the activation of protein kinase C family members and their downstream targets such as MAPK, thus affecting cell proliferation, apoptosis, and differentiation. **Panel B. **Since HSL is the key neutral cholesteryl ester hydrolase in adipose tissue, a regulatory pool of free cholesterol might be depleted in HSL null mice leading to an increase in the transcription factor SREBP2 (sterol regulatory element binding protein 2) and a subsequent up-regulation of its transcriptional targets such as UCP2 (uncoupling protein 2). **Panel C. **Since HSL mediates the mobilization of fatty acids, the release of specific fatty acids by HSL might be required for the production of ligands that are preferentially utilized by PPARγ. A relative lack of PPARγ ligands might suppress the mutual activation of PPARγ and C/EBPα, the 2 most important transcription factors required for adipocyte differentiation.

HSL null mice display defective lipolysis due to diminished expression of lipases and lipid droplet-associated proteins, reduced quantities of WAT with increased amounts of BAT, increased numbers of macrophages in WAT, are resistant to high-fat diet induced obesity secondary to an apparent increase in thermogenesis and energy expenditure, and have multiple alterations in the expression of genes involved in adipose differentiation, including transcription factors, markers of adipocyte differentiation, and enzymes of fatty acid and triglyceride synthesis. Although the mechanisms responsible for these alterations are not clear, at least three possibilities in addition to infiltration of macrophages might explain these findings. First, since HSL is apparently the primary diacylglycerol lipase in adipose tissue, the accumulation of diacylglycerol in WAT of HSL null mice might interfere with normal adipocyte differentiation or function through the activation of protein kinase C family members and their downstream targets, which are known to affect cell proliferation, apoptosis, and differentiation (Figure 2A). Second, since HSL appears to be the only neutral cholesteryl ester hydrolase in adipose tissue, the inability to hydrolyze cholesteryl esters might reduce regulatory pools of cellular unesterified cholesterol (Figure 2B), which has been suggested to be a sensor linking adipose cell size to metabolism [24]. This might lead to an up-regulation of SREBP2 and a subsequent increase in UCP2. Third, since HSL mediates the mobilization of fatty acids, the release of specific fatty acids by HSL might be required for the production of ligands that are preferentially utilized by PPARγ (Figure 2C). A relative lack of PPARγ ligands due to the absence of HSL might suppress the mutual activation of PPARγ and C/EBPα, thus affecting adipocyte differentiation. Whichever the molecular basis for these alterations, HSL null mice have proven to be extremely useful in helping to elucidate the pathways of lipolysis and an important model for studying adipose cell metabolism.

## List of abbreviations

Free fatty acids, FFA; hormone-sensitive lipase, HSL; protein kinase A, PKA; brown adipose tissue, BAT; white adipose tissue, WAT; adipose triglyceride lipase, ATGL; peroxisome proliferators activated receptor, PPAR; CCAAT/enhancer binding protein, C/EBP; tumor necrosis factorα, TNFα; glycerol-3-phosphate acyltransferase, GPAT; acyl-CoA:diacylglycerol acyltransferase, DGAT; 3-hydroxy-3-methylglutaryl-CoA, HMG CoA; sterol regulatory element binding protein, SREBP.

## Competing interests

The author(s) declare that they have no competing interests.

## Authors' contributions

FBK drafted the manuscript. WJS carried out some of the original experiments described.
